# Risk Factors for Perioperative Brain Lesions in Infants With Congenital Heart Disease: A European Collaboration

**DOI:** 10.1161/STROKEAHA.122.039492

**Published:** 2022-10-27

**Authors:** Alexandra F. Bonthrone, Raymond Stegeman, Maria Feldmann, Nathalie H.P. Claessens, Maaike Nijman, Nicolaas J.G. Jansen, Joppe Nijman, Floris Groenendaal, Linda S. de Vries, Manon J.N.L. Benders, Felix Haas, Mirielle N. Bekker, Thushiha Logeswaran, Bettina Reich, Raimund Kottke, Cornelia Hagmann, Beatrice Latal, Hitendu Dave, John Simpson, Kuberan Pushparajah, Conal Austin, Christopher J. Kelly, Sophie Arulkumaran, Mary A. Rutherford, Serena J. Counsell, Walter Knirsch, Johannes M.P.J. Breur

**Affiliations:** Centre for the Developing Brain, School of Biomedical Engineering and Imaging Sciences, King’s College London, United Kingdom (A.F.B., K.P., C.J.K., S.A., M.A.R., S.J.C.).; Department of Neonatology (R.S., N.H.P.C., M.N., F.G., L.S.d.V., M.J.N.L.B.), University Medical Center Utrecht, Utrecht University, the Netherlands.; Department of Pediatric Intensive Care (N.H.P.C., M.N., N.J.G.J., J.N.), University Medical Center Utrecht, Utrecht University, the Netherlands.; Department of Pediatric Cardiology (N.H.P.C., M.N., J.M.P.J.B.), University Medical Center Utrecht, Utrecht University, the Netherlands.; Congenital Cardiothoracic Surgery (F.H.), University Medical Center Utrecht, Utrecht University, the Netherlands.; Department of Obstetrics (M.N.B.), University Medical Center Utrecht, Utrecht University, the Netherlands.; Wilhelmina Children’s Hospital and Utrecht Brain Center (R.S., N.H.P.C., M.N., F.G.), University Medical Center Utrecht, Utrecht University, the Netherlands.; Child Development Center (M.F., B.L.), University Children’s Hospital Zurich, Switzerland.; Department of Diagnostic Imaging (R.K.), University Children’s Hospital Zurich, Switzerland.; Department of Neonatology and Pediatric Intensive Care (C.H.), University Children’s Hospital Zurich, Switzerland.; Department of Pediatrics, Beatrix Children’s Hospital, University Medical Center Groningen, the Netherlands (N.J.G.J.).; Pediatric Heart Center, University Hospital Giessen, Justus-Liebig-University Giessen, Germany (T.L.).; Department of Congenital Heart Disease and Pediatric Cardiology, German Heart Center Munich, Technical University of Munich, Germany (B.R.).; Division of Congenital Cardiovascular Surgery (H.D.), Pediatric Heart Center, Department of Surgery, Children’s Research Center, University Children’s Hospital Zurich, University of Zurich, Switzerland.; Pediatric Cardiology (W.K.), Pediatric Heart Center, Department of Surgery, Children’s Research Center, University Children’s Hospital Zurich, University of Zurich, Switzerland.; Pediatric Cardiology Department, Evelina Children’s Hospital London, United Kingdom (J.S., K.P., C.A.).

**Keywords:** heart diseases, ischemic stroke, magnetic resonance imaging, venous thrombosis, pedatrics, risk factors, white matter

## Abstract

Infants with congenital heart disease are at risk of brain injury and impaired neurodevelopment. The aim was to investigate risk factors for perioperative brain lesions in infants with congenital heart disease.

**Methods:**

Infants with transposition of the great arteries, single ventricle physiology, and left ventricular outflow tract and/or aortic arch obstruction undergoing cardiac surgery <6 weeks after birth from 3 European cohorts (Utrecht, Zurich, and London) were combined. Brain lesions were scored on preoperative (transposition of the great arteries N=104; single ventricle physiology N=35; and left ventricular outflow tract and/or aortic arch obstruction N=41) and postoperative (transposition of the great arteries N=88; single ventricle physiology N=28; and left ventricular outflow tract and/or aortic arch obstruction N=30) magnetic resonance imaging for risk factor analysis of arterial ischemic stroke, cerebral sinus venous thrombosis, and white matter injury.

**Results:**

Preoperatively, induced vaginal delivery (odds ratio [OR], 2.23 [95% CI, 1.06–4.70]) was associated with white matter injury and balloon atrial septostomy increased the risk of white matter injury (OR, 2.51 [95% CI, 1.23–5.20]) and arterial ischemic stroke (OR, 4.49 [95% CI, 1.20–21.49]). Postoperatively, younger postnatal age at surgery (OR, 1.18 [95% CI, 1.05–1.33]) and selective cerebral perfusion, particularly at ≤20 °C (OR, 13.46 [95% CI, 3.58–67.10]), were associated with new arterial ischemic stroke. Single ventricle physiology was associated with new white matter injury (OR, 2.88 [95% CI, 1.20–6.95]) and transposition of the great arteries with new cerebral sinus venous thrombosis (OR, 13.47 [95% CI, 2.28–95.66]). Delayed sternal closure (OR, 3.47 [95% CI, 1.08–13.06]) and lower intraoperative temperatures (OR, 1.22 [95% CI, 1.07–1.36]) also increased the risk of new cerebral sinus venous thrombosis.

**Conclusions:**

Delivery planning and surgery timing may be modifiable risk factors that allow personalized treatment to minimize the risk of perioperative brain injury in severe congenital heart disease. Further research is needed to optimize cerebral perfusion techniques for neonatal surgery and to confirm the relationship between cerebral sinus venous thrombosis and perioperative risk factors.

Congenital heart disease (CHD) requiring intervention in early infancy occurs in ≈0.3% of live births.^1^ Up to 90% of children live into adulthood,^2^ however, survivors are at increased risk of neurodevelopmental impairments.^3,4^ Research has increasingly focused on understanding mechanisms underlying impaired neurodevelopment in CHD.

Magnetic resonance imaging (MRI) studies have identified brain injuries in infants with CHD before and after cardiac surgery.^5–7^ White matter injury (WMI) and arterial ischemic stroke (AIS) are most commonly reported,^8,9,11^ however, cerebral sinus venous thrombosis (CSVT), hypoxic-ischemic watershed injury, and intraparenchymal hemorrhage are also observed.^12,13^

Risk factor analyses have implicated birth history,^14,15^ clinical course,^14,16–20^ catheterization and surgical procedures^11,14,18,21–24^ and cardiac diagnosis^11,18,19,25^ in perioperative brain injury in CHD. Many previous studies combined infants with different injuries, it is, therefore, unclear if identified factors are common to all injuries or specific to certain forms.

Here, we assessed risk factors for preoperative and new postoperative AIS, CSVT, and WMI, due to their common occurrence and potential impact on neurodevelopment, in infants with CHD requiring cardiac surgery <6 weeks after birth.^13^

## Methods

### Data Availability

The data that support the findings of this study are available from the corresponding author upon reasonable request.

### Recruitment

**Figure 1.**
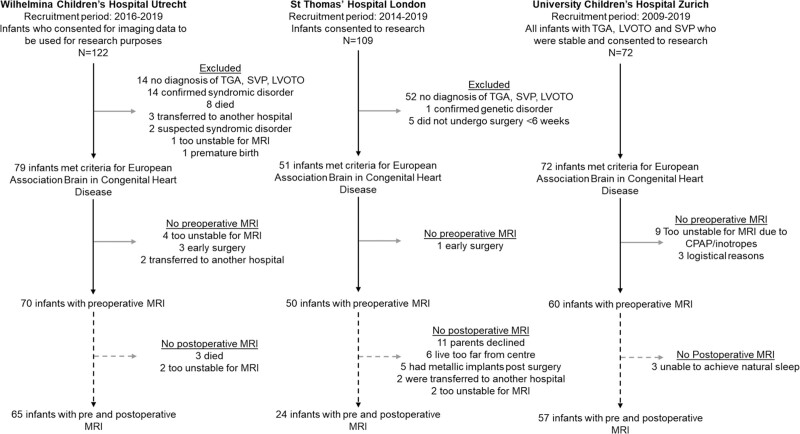
**Flow diagram showing inclusion and exclusion of infants from each research center.** CPAP indicates continuous positive airway pressure; LVOTO, left ventricular outflow tract and/or aortic arch obstruction; MRI, magnetic resonance imaging; SVP, single ventricle physiology; and TGA, transposition of the great arteries.

One hundred eighty infants with CHD (Table 1) who underwent cardiac surgery <6 weeks after birth were prospectively recruited in 3 observational cohort studies at Wilhelmina Children’s Hospital Utrecht, University Children’s Hospital Zurich, and St Thomas’ Hospital London and combined (see Figure 1 for recruitment details). Infants with transposition of the great arteries (TGA, with/without arch obstruction), single ventricle physiology (SVP), or left ventricular outflow tract and/or aortic arch obstruction, such as aortic valve stenosis, hypoplastic left heart complex or aortic coarctation, hypoplasia or interruption (left ventricular outflow tract and/or aortic arch obstruction), were included. Infants with known/suspected genetic/syndromic disorders were excluded. Brain MRI was performed preoperatively and postoperatively per clinical (Utrecht) or research (Zurich and London) protocol. One hundred forty-six infants underwent postoperative MRI (Table 2).

**Table 1.**
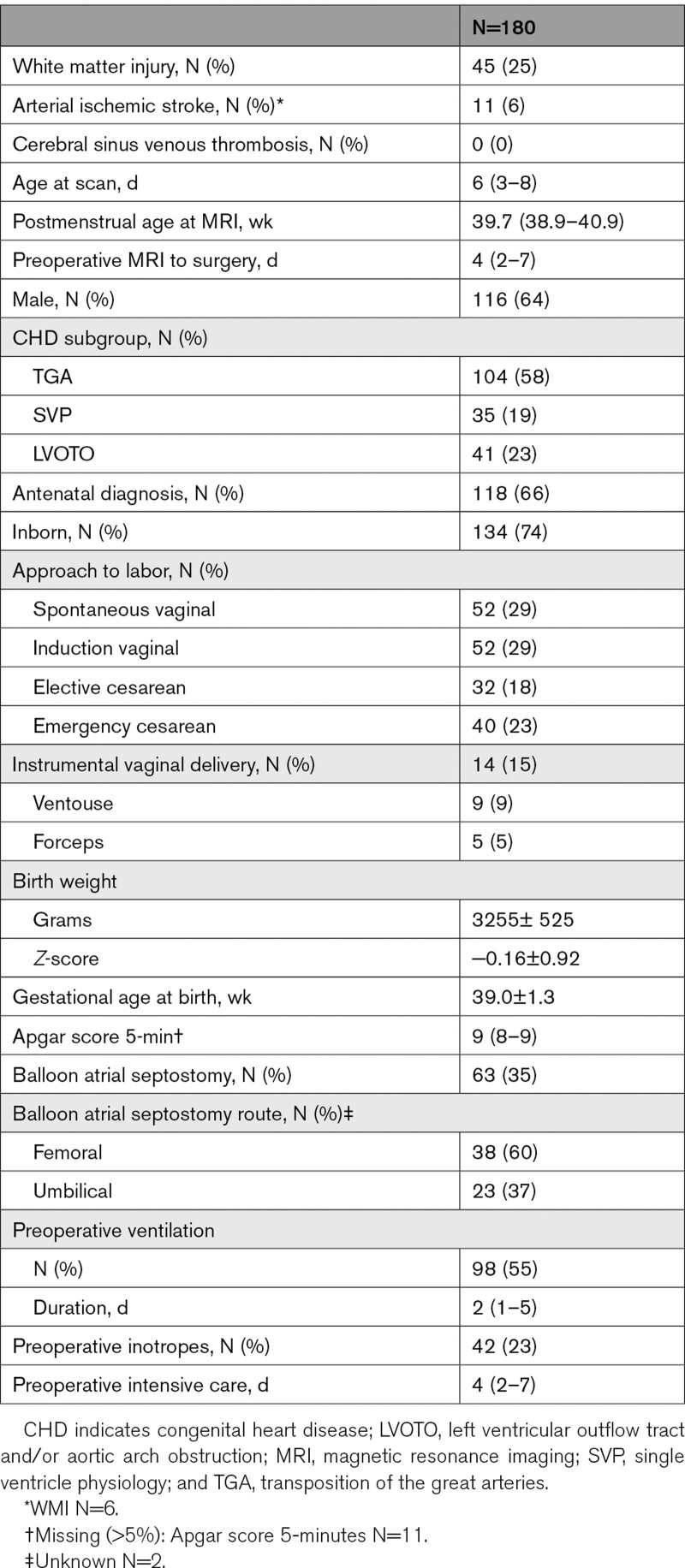
Clinical Characteristics of Infants Who Underwent Preoperative MRI

**Table 2.**
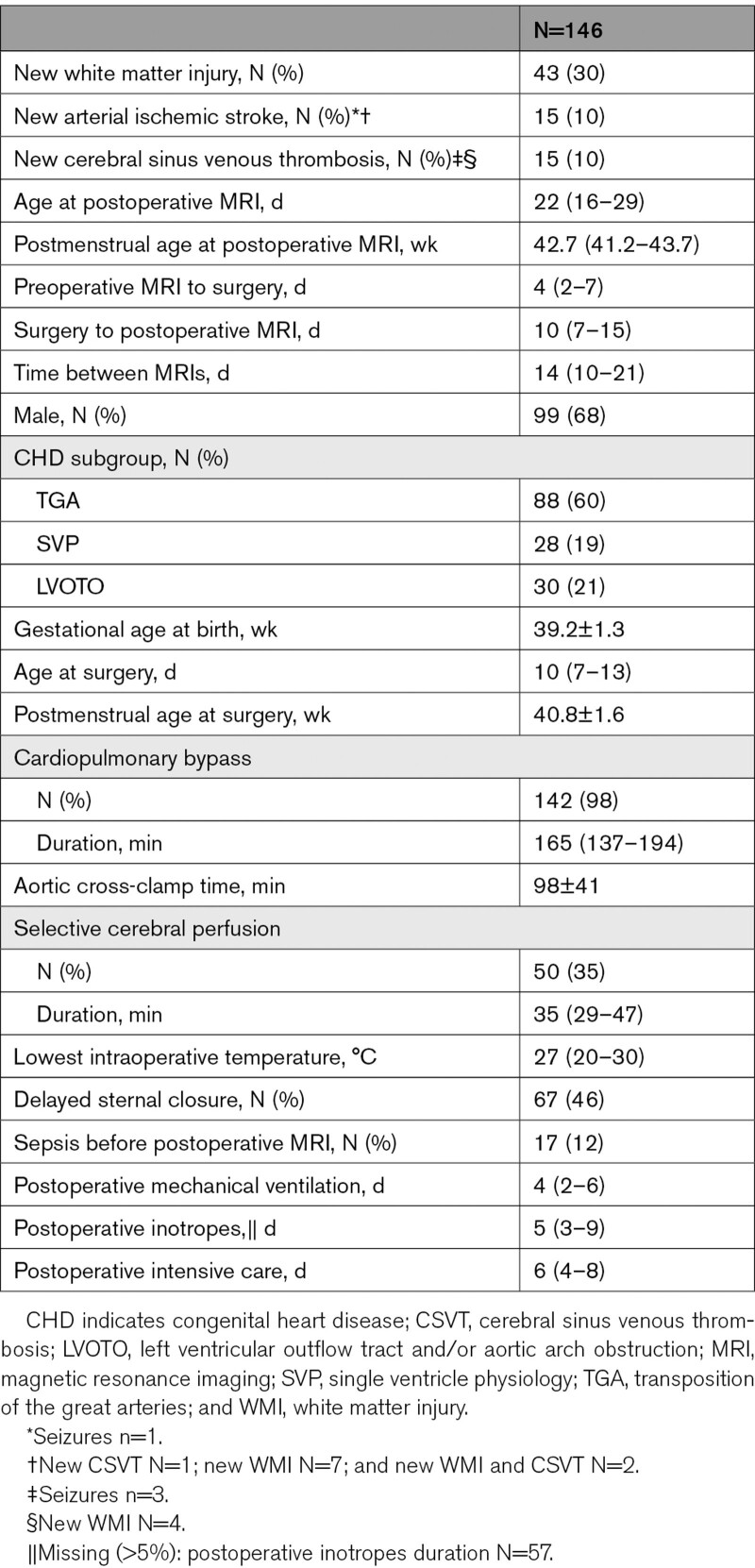
Perioperative Clinical Characteristics of Infants Who Underwent Preoperative and Postoperative MRI

Clinical characteristics were collected at each center and combined (see Tables S1 and S2 for additional characteristics). Respective institutional research ethics committees provided approval (Utrecht, no. 16-093; Zurich KEK StV-23/619/04; and London 07/H0707/105). Parental informed consent was obtained for use of clinical data for research purposes (Utrecht) or before study enrollment (Zurich and London). This article follows STROBE reporting guidelines (https://www.strobe-statement.org/, Supplemental Material).

### MRI Protocol and Image Review

All infants underwent 3-Tesla brain MRI. MRI system, head-coil, scanning procedures, protocols, and image review procedures are described in Stegeman et al.^13^ Briefly, all infants underwent T1-, T2-, and diffusion-weighted imaging. Utrecht and London also acquired MR venography and susceptibility-weighted imaging. Zurich acquired venography and susceptibility-weighted imaging when there was evidence of CSVT or hemorrhage on conventional imaging.

A consensus on terminology, definition and scoring of brain MRI was achieved in a joint MRI meeting. Zurich, London, and Utrecht applied a uniform description of brain imaging findings described previously,^13^ which was adapted from work by Beca et al,^11^ to MRI scans from their local cohorts. Brain MRI findings are summarized in Tables 1 and 2, see Table S3 for information on location of injuries.

Postoperative brain MRI findings were considered new if preoperative MRI showed no corresponding findings, lesion(s) location(s) were different, and size or number increased.

Presence/absence of WMI, AIS, and CSVT on preoperative/new postoperative MRI were primary outcome variables.

### Statistical Analysis

Analyses were performed using SPSS (V.25.0) and R (V.3.6.2). Normality was assessed with histograms, Q-Q plots, and Kolmogorov-Smirnov/Shapiro-Wilk tests. Birth weight was transformed into *Z* scores with the United Kingdom-World Health Organisation reference data.^26^ Continuous data are presented as mean±SD when normally distributed or as median (interquartile range) when not. Categorical data are presented as N (%).

Potential risk factors were selected based on previous literature. We examined factors related to infant demographics,^11,18,27^ pregnancy and delivery,^15,16,27^ intensive care,^14,18,21,22^ perioperative course,^8,11,14,18,24,28^ and MRI^11,29^ (see Table 3 for risk factors assessed). Differences between infants with and without preoperative/new postoperative WMI, AIS, and CSVT were assessed using independent t test, Mann-Whitney *U*, χ^2^, and Fisher exact.

**Table 3.**
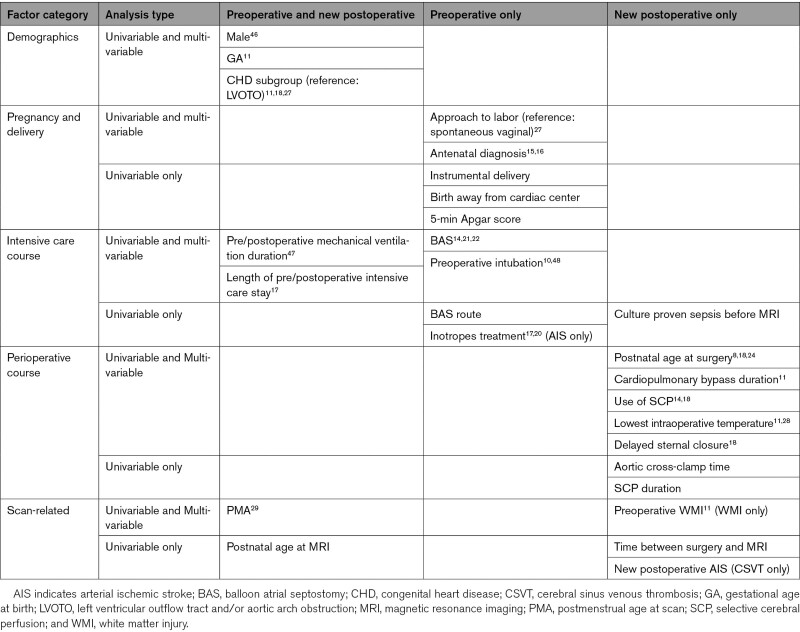
Clinical Variables Included in Statistical Analyses Based on Previous Literature

*P* values underwent false discovery rate correction (*P*_FDR_) to correct for multiple comparisons. *P*_FDR_<0.05 was considered statistically significant. Significant results are presented as percentages or median (95% ) difference.

Relationships between clinical variables were assessed by 1-way analysis of variance, Kruskal-Wallis, χ^2^, Fisher exact, and Pearson correlations.

Multivariable backward stepwise logistic regression (exclusion criteria *P*>0.1) was used to identify risk factors for preoperative/new postoperative WMI, AIS, and CSVT. Variables were selected based on previous literature (Table 3) and results of univariable analyses.

Pearson correlations were used to test for multicollinearity. Preoperative postmenstrual age at scan (PMA) and gestational age at birth (GA; *r*=0.909), cardiopulmonary bypass duration and aortic cross-clamp time (*r*=0.845), and postoperative ventilation duration and intensive care stay (*r*=0.719) were collinear. GA, cardiopulmonary bypass duration, and ventilation duration were included unless alternate variables were significant in univariable analyses.

Results were presented as adjusted odds ratios and absolute risks (95% CI).

Sensitivity analysis was performed by repeating analyses for new postoperative injuries excluding infants who did not undergo cardiopulmonary bypass (arch repair n=2, hybrid procedure n=1; and missing data aortopulmonary shunt n=1).

## Results

### Univariable Differences

New postoperative AIS was associated with younger age at surgery (7 [6–9] versus 11 [7–15] days; *P*_FDR_=0.019), selective cerebral perfusion (SCP; 80% versus 29%; *P*_FDR_=0.003), lower intraoperative temperatures (19.7 °C [19.0–27.5] versus 27.4 °C [21.6–30.0]; *P*_FDR_=0.048), longer mechanical ventilation (6 [4–15] versus 3 [2–5] days; *P*_FDR_=0.004), longer postoperative intensive care stay (11 [8–30] versus 6 [4–8] days; *P*_FDR_=0.019), and CHD subgroup (SVP 53%; TGA 27%; and left ventricular outflow tract and/or aortic arch obstruction 20%; *P*_FDR_=0.007; Table S4).

Infants with preoperative AIS had lower 5-minute Apgar scores (8 [6–8]) than infants without (9 [8–9]; *P*_FDR_=0.019; Table S5).

There were no significant differences between infants with and without preoperative/new postoperative WMI or new postoperative CSVT (Tables S6 through S8).

### Multivariable Logistic Regressions

#### Preoperative Brain Injuries

**Figure 2.**
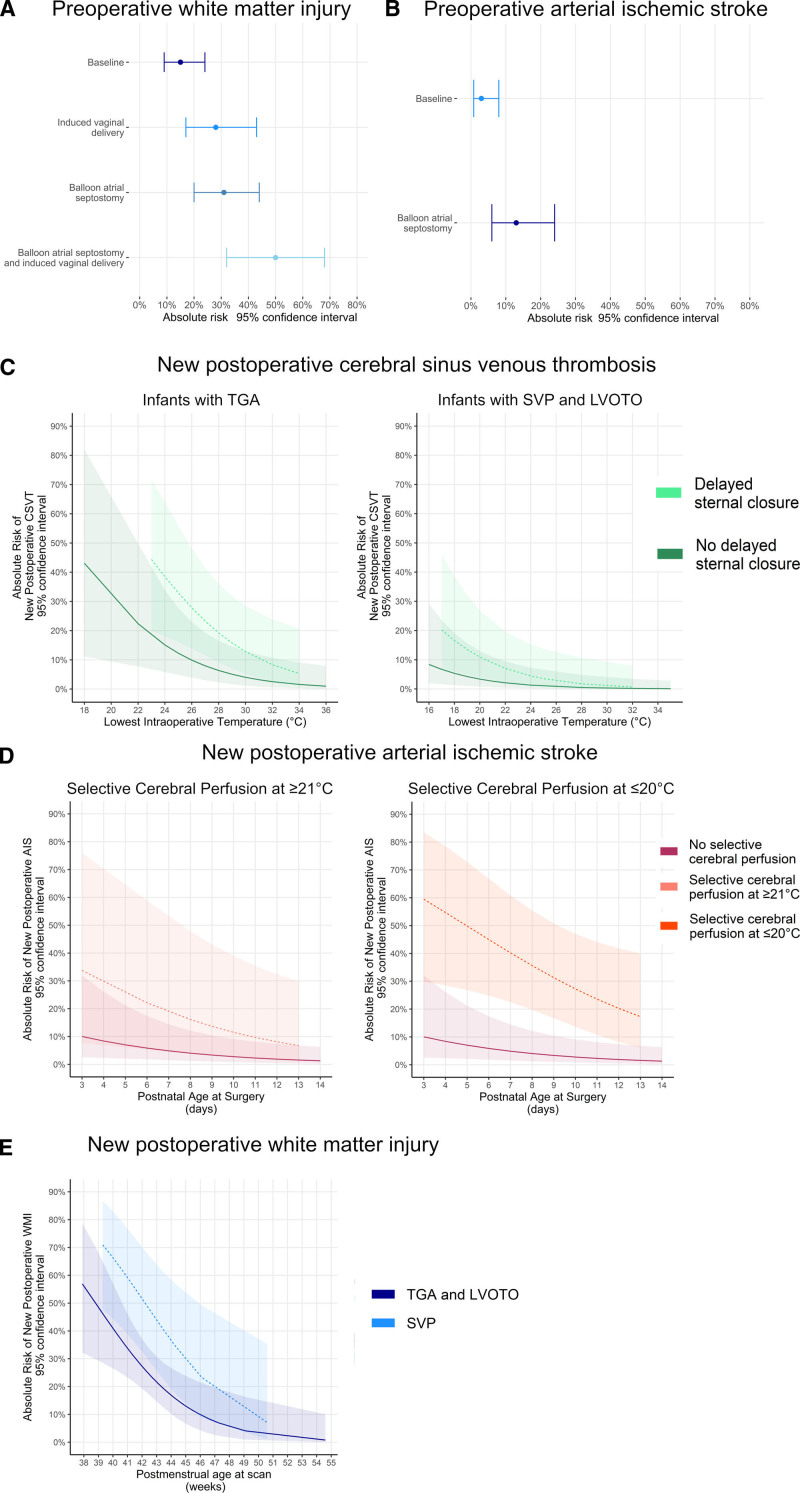
**Risk factors and absolute risk (95%CI) for perioperative brain lesions in infants with congenital heart disease.** Risk factors for preoperative (**A**) white matter injury, (**B**) arterial ischemic stroke; and new postoperative (**C**) cerebral sinus venous thrombosis, (**D**) arterial ischemic stroke, and (**E**) white matter injury. CSVT indicates cerebral sinus venous thrombosis; LVOTO, left ventricular outflow tract and/or aortic arch obstruction; SVP, single ventricle physiology; and TGA, transposition of the great arteries.

Induced vaginal delivery and balloon atrial septostomy (BAS) increased the risk of preoperative WMI (Table 4, Figure 2A). Infants born by induced vaginal delivery had lower GA compared with spontaneous vaginal delivery (*P*=0.007) but not elective (*P*=0.087) or emergency (Table S9; *P*=0.861) cesarean section, however, including GA in the final model did not change the results (Table 4).

**Table 4.**
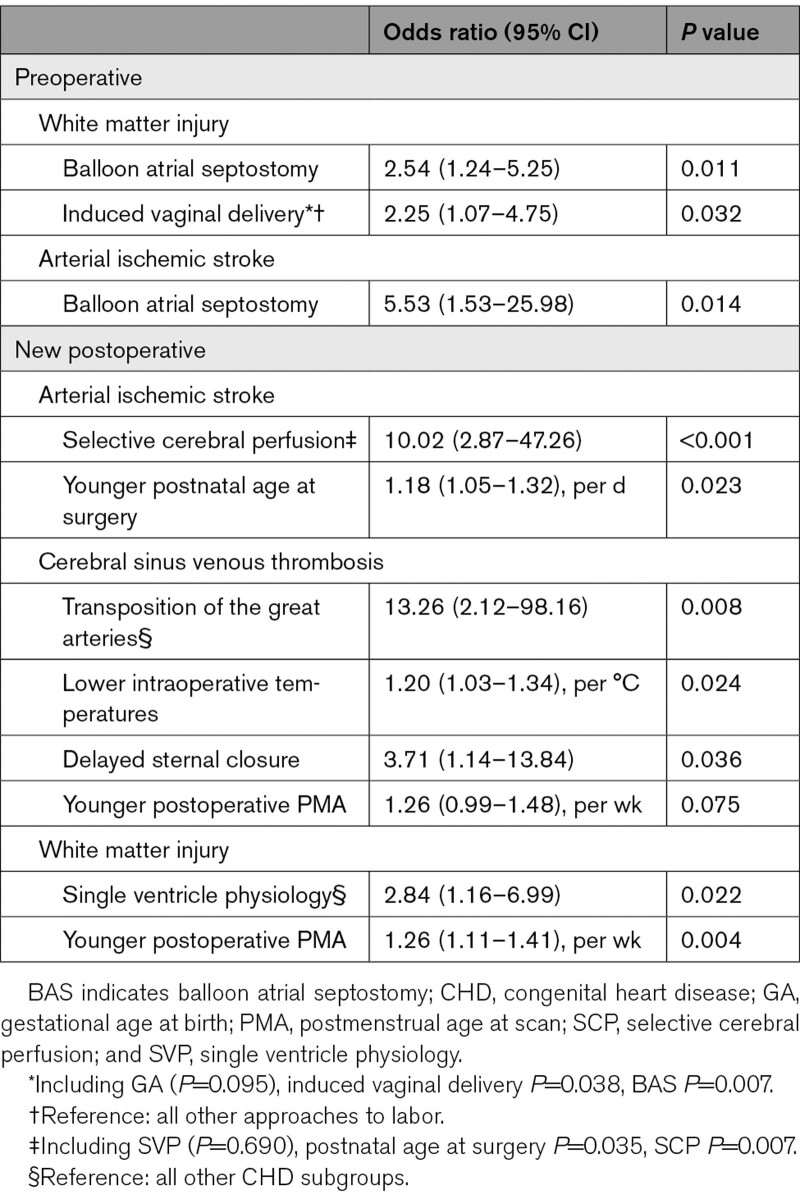
Multivariable Regression Analyses of Perioperative Brain Injuries

The proportion of infants with WMI was not different between infants induced for CHD alone (11 of 37 infants; 29.7%) and infants induced for additional reasons (5 of 13 infants; 38.4%, *P*=0.731; Table S9).

BAS was associated with preoperative AIS (Table 4; Figure 2B).

### New Postoperative Brain Injuries

SCP and younger postnatal age at surgery were associated with new postoperative AIS (Table 4); predicting right-sided (n=10; age at surgery *P*=0.024; SCP *P*=0.007) but not left sided AIS (n=4; age at surgery *P*=0.512; SCP *P*=0.129).

Postnatal age at surgery was associated with CHD subgroup (Table S2, *P*=0.007), however, including SVP in the final model did not change the results (Table 4). There were no new postoperative AIS in infants operated after 13 days. Preoperative mechanical ventilation (median interquartile range not ventilated 9 [3–41], ventilated 11 [3–42]; *P*=0.004) and treatment with inotropes (no treatment median 9 [3–42], treatment median 12.5 interquartile range [4–41]; *P*=0.008) were associated with later age at surgery.

Post hoc regressions with SCP split into 4 categories: no SCP, SCP at >28 °C, SCP at 21 to 28 °C, and SCP at ≤20 °C (Table S10) revealed SCP at ≤20 °C was associated with new postoperative AIS (odds ratio, 13.46 [3.58–67.10]; *P*<0.001; Figure 2D).

TGA, lower intraoperative temperatures, and delayed sternal closure were risk factors for new postoperative CSVT (Table 4; Figure 2D). TGA remained a significant predictor (odds ratio, 46.66 [3.40–893]; *P*=0.003) when controlling for arch repair. No infants with new postoperative CSVT had venous infarcts, see Table S11 for details.

SVP and younger postoperative PMA were risk factors for new postoperative WMI (Table 4; Figure 2E). PMA was unrelated to CHD subgroup (Table S2; *P*=0.256).

### Sensitivity Analysis

Univariable (Tables S4, S7, and S8) and multivariable (Table S12) results were not different when excluding infants who did not undergo cardiopulmonary bypass.

## Discussion

This study investigated risk factors for perioperative brain injuries in infants with CHD. Younger postnatal age at surgery and SCP during surgery, particularly at ≤20 °C, increased the risk of new postoperative AIS. Infants with SVP were at highest risk of new WMI while those with TGA were at risk of new CSVT. Lower intraoperative temperatures and delayed sternal closure were also risk factors for new CSVT. Preoperatively, induced vaginal delivery was associated with WMI and BAS was associated with brain injury. These results present novel opportunities for personalized treatment to minimize the burden of perioperative brain injury in neonates with CHD.

Induced vaginal delivery was associated with preoperative WMI. Interestingly, the occurrence of WMI was not different between infants induced for CHD alone and those induced for additional reasons. In contrast, Kelly et al^7^ reported no significant relationship between induction of delivery and preoperative WMI. Their study examined the impact of induction regardless of delivery method in a wider range of CHD diagnoses, which may account for differing results. It is unclear how induced vaginal delivery might increase risk of WMI. Type of delivery has no reported impact on neonatal stability in CHD.^30^ In healthy infants, induction is not associated with longer duration of labor^31^ and evidence of increased oxidative stress after uncomplicated vaginal delivery is mixed,^32^ although to our knowledge this has not been investigated in CHD. WMI occurs in around 12% of otherwise healthy infants scanned for research purposes,^33^ most commonly in infants delivered by ventouse, however, the impact of induced vaginal delivery has not been investigated. In our centers, mothers who live locally await spontaneous labor whereas those who do not are induced at 38 or 39 weeks where possible, however, differences in GA did not explain the association with WMI. Our results suggest induced vaginal delivery may be associated with WMI in infants who are susceptible to brain injury, however further research is needed. It is possible that infants with CHD, particularly those requiring BAS, may benefit from delivery by alternate strategies to reduce the cumulative risk of preoperative WMI. However, alternate delivery plans are not without risks to both infants and mothers. Nevertheless, our results may provide novel opportunities to individualize care of infants with CHD.

In agreement with several studies, BAS, but not route of access, was associated with preoperative brain injury.^7,14,18,20,22^ BAS is used to improve systemic oxygen saturation, primarily in neonates with TGA. Routine BAS in TGA regardless of hemodynamic stability has been associated with increased prevalence of preoperative focal ischemic lesions.^18^ In our centers, BAS is performed in infants with unacceptably low preductal oxygen saturations. Therefore, infants requiring BAS are already exposed to hypoxemia and potential cardiovascular disruption which may increase vulnerability to brain injury.^7,22^ Indeed, infants with preoperative AIS had lower 5-minute Apgar scores. Earlier cardiac surgery may reduce the need for BAS. However, cardiac surgery in unstable infants is inherently risky, and we also identified younger age at surgery as a risk factor for postoperative AIS. These competing risks should be considered in individual infants.

This study did not investigate different intraoperative perfusion techniques; however, we identified an association between SCP at deep hypothermia and new postoperative AIS. Optimal protocols for neonatal SCP are controversial and a systematic review did not find enough evidence to recommend a perfusion or cooling strategy.^31^ There is emerging evidence that mild or moderate hypothermia during SCP may be optimal for neonatal neuroprotection.^32^ In piglet models, SCP at 25 to 27 °C maintained brain glucose levels^33^ and cerebral oxygenation was comparable to lower temperatures.^34^ In a small infant study, SCP at 23 to 25 °C was protective for new postoperative ischemic lesions compared with deep hypothermic circulatory arrest.^34^ Randomized controlled trials report no differences in new postoperative brain injury incidence and neurodevelopmental outcomes in infants who undergo SCP at 18 °C compared with deep hypothermic circulatory arrest.^35,36^ SCP was also previously identified as a risk factor for new postoperative focal ischemic injury in CHD.^18^ As our multi-center study was observational, it may be detrimental to draw conclusions about the merits of SCP. However, our findings support calls to optimize SCP protocols in neonates.^34,37,38^

Younger postnatal age at surgery was a risk factor for new postoperative AIS. Previous findings are inconsistent, with both younger^8^ and older age at surgery^24^ associated with postoperative WMI. It was recently reported that risk of new postoperative ischemic injury increases from birth to 9 days at surgery before decreasing to a minimum at around 27 days.^18^ Earlier surgery has been associated with better clinical outcomes and lower health care costs^39^ and better language abilities at 18 months.^40^ Interestingly, in this cohort, infants operated at a later age were more likely to be ventilated and treated with inotropes before surgery, perhaps reflecting more severe illness. Later surgery may be an important tool for minimizing the risk of AIS, particularly in infants undergoing SCP or those with preoperative injury.

There is emerging evidence that different cardiac physiologies increase the risk of distinct postoperative brain injuries. We identified infants with TGA as at risk of new CSVT and supported other studies reporting infants with SVP are at risk of new WMI.^11,25^ Identifying cardiac physiologies as initial risk factors for new postoperative injury allow individualized perioperative care to reduce brain injury.

To our knowledge, this is the first study to identify delayed sternal closure and lower intraoperative temperatures as risk factors for new postoperative CSVT. One previous study reported no differences in clinical characteristics between infants with and without new postoperative CSVT.^12^ Delayed sternal closure is used to prevent hemodynamic instability postsurgery.^41^ These infants are, therefore, the most complex, requiring prolonged surgical procedures and intensive care stays. Lower intraoperative temperatures increase the risk of excessive postoperative bleeding in infants undergoing CPB^42^ requiring perioperative coagulatory therapy. The significance of these factors is difficult to assess, therefore, further large studies are needed to investigate perioperative risk factors for CSVT.

Younger postoperative PMA increased the odds of identifying new postsurgical WMI. In premature infants, some WMI identified on early MRI are not visible at term-equivalent age.^29^ Some infants with CHD may have had new WMI that resolved before postoperative MRI. Nevertheless, PMA was not different between CHD subgroups and SVP remained a significant predictor of new WMI when controlling for PMA.

### Limitations and Future Directions

We acknowledge that this work has some limitations. We retrospectively combined data from 3 European centers and the proportion of SVP differed between cohorts (66%, 22%, and 12%). We therefore did not examine differences in lesion incidence, or perinatal, perioperative, and surgical management between centers. Nevertheless, by combining data across 3 centers, this study included a large cohort of infants with CHD with comparable MRI and clinical details.

This observational study investigated multiple inter-related risk factors. We could not definitively disentangle how perioperative factors, CHD diagnosis, and preoperative brain injury might interact. We, therefore, do not draw any conclusions about optimized neuroprotection during surgery. Further discussion is warranted regarding the feasibility of randomized controlled trials to definitively determine if different approaches to labor, intraoperative neuroprotective techniques or timing of surgery reduce the prevalence of brain injury in neonates with CHD.

MR venography was not routinely performed in Zurich which may have led to an underestimation of the prevalence of CSVT.

Previous studies have suggested that moderate/severe WMI in infancy is associated with neurodevelopmental impairments in childhood.^25,43^ Similarly, the neurodevelopmental consequences of AIS are likely dependent on factors such as volume and location.^44,45^ Future studies should investigate the associations between perioperative factors, cumulative burden, location and size of injuries, and neurodevelopmental outcomes. Nevertheless, it is important to consider risk factors for presence of perioperative injury, so that care may be tailored to minimize the cumulative burden.

### Conclusions

This study identified risk factors for perioperative brain injury in infants with CHD introducing novel opportunities for tailored perinatal and perioperative care based on risk stratification. Further research is needed to optimize SCP protocols for neonates and to confirm the relationship between CSVT and perioperative risk factors.

## Article Information

### Acknowledgments

This work was conducted as part of the European Association Brain in Congenital Heart Disease (European ABC) Consortium. Our thanks go to the families who participated in this research. We also thank Prof Dr Matthias Müller from the Pediatric Heart Center at Giessen and Dr Monique Haak from Leiden UMC for their valuable feedback. We thank, from Utrecht: the Congenital Heart Disease LifeSpan Research group, which includes staff from the Departments of Obstetrics, Pediatric Heart Center, Pediatric Intensive Care, Neonatology, Center for Child Development, Exercise and Physical Literacy, and Radiology. From London: staff from the St Thomas’ Neonatal Intensive Care Unit; Evelina London Fetal and Paediatric Cardiology Departments and Paediatric Intensive Care Unit; Centre for the Developing Brain at King’s College London; our research radiographers and neonatal scanning team. From Zurich: Dr Ruth O’Gorman Tuura for the set up and technical supervision of MR scanning protocols; staff from the Center for MR Research; Pediatric Heart Center; Department of Intensive Care Medicine and Neonatology; Department of Diagnostic Imaging at the University Children’s Hospital Zurich.

### Sources of Funding

This research was funded by a Consolidator Grant of the European Society of Paediatric Research. Utrecht: The Hartekind Foundation and Vrienden van het Wilhelmina Kinderziekenhuis Foundation and by ZONMW (doelmatigheidsonderzoek) Crucial trial. London: Medical Research Council UK (MR/V002465/1; MR/L011530/1), Action Medical Research (GN2630), and the British Heart Foundation (FS/15/55/31649) grants awarded to Prof Counsell, supported by core funding from the Wellcome/EPSRC Centre for Medical Engineering (WT203148/Z/16/Z) and the National Institute for Health Research (NIHR) Biomedical Research Centre based at Guy’s and St Thomas’ National Health Service (NHS) Foundation Trust and King’s College London. The views expressed are those of the author(s) and not necessarily those of the NHS, the NIHR or the Department of Health and Social Care. Zurich: Mäxi-Foundation, Anna Müller Grocholski Foundation, and EMDO Foundation Zurich.

### Disclosures

Dr Groenendaal is co-inventor of 2-Iminobiotin as a neuroprotective agent in neonates with hypoxic-ischemic encephalopathy. Prof Benders is a consultant for neonatal brain injury for Chiesi pharmaceuticals. The other authors report no conflicts.

### Supplemental Material

STROBE Checklist

Tables S1–12

## Supplementary Material


