# Supplementary material for: Risk Factors for Perioperative Brain Lesions in Infants with Congenital Heart Disease: A European Collaboration
Source: Stroke. Author manuscript; Available in PMC 2022 Dec 1. (PMC9698124; doi:10.1161/STROKEAHA.122.039492)
Supplement: Supplementary material 1 [file EMS155068-supplement-Supplementary_material_1.pdf]

Table S1. Clinical characteristics of infants who underwent preoperative MRI by CHD subgroup				
	**Transposition of the Great Arteries N=104**	**Single Ventricle Physiology N=35**	**Left ventricular outflow tract and/or aortic arch obstruction N=41**	**p_{FDR}**
**Age at preoperative scan**, days	6 (4-7)	4 (2.5-6.5)	5 (2-8)	0.180
**Postmenstrual age at preoperative scan**, weeks	40.1 (39.2-41.0)	39.6 (38.6-40.0)	39.4 (38.7-40.9)	0.122
**Time preoperative MRI to surgery**, days	4.5 (2-8)	3 (1-4.5)	4 (3-6)	0.152
**Male**, N (%)	67 (64)	22 (63)	27 (66)	1.00
**Antenatal diagnosis**, N (%)	57 (55)	31 (89)	30 (73)	**0.004**
**Born at cardiac center**, N (%)	70 (67)	31 (89)	33 (80)	0.101
**Approach to labor**, N (%)				
Spontaneous vaginal	35 (34)	6 (17)	11 (27)	0.501
Induction vaginal	28 (27)	14 (40)	10 (24)	
Elective cesarean	15 (14)	8 (23)	9 (22)	
Emergency cesarean	25 (24)	6 (17)	9 (22)	

**Instrumental vaginal delivery**, N (%)<br>Ventouse<br>Forceps	8 (8)<br>5 (5)<br>3 (3)	1 (3)<br>1 (3)<br>0	5 (12)<br>3 (7)<br>2 (5)	0.501
**Birth weight**<br>Grams<br>Z-score	3300 (2948-3673)<br>-0.11 (-0.80 – 0.58)	3160 (2945-3423)<br>-0.28 (-0.71 - 0.14)	3135 (2860-3765)<br>-0.19 (-0.94-0.56)	0.501<br>0.603
**Gestational age at birth**, weeks	39.1 (38.6-40.2)	39 (38.1-39.3)	38.7 (38-40)	0.180
**Twin**, N (%)	4 (4)	1 (3)	1 (2)	1.00
**Apgar score 5-minutes***	9 (8-9)	9 (9-10)	9 (9-10)	**<0.001**
**Arterial cord pH at birth***	7.29 (7.24-7.35)	7.26 (7.18-7.35)	7.28 (7.21-7.36)	1.00
**Head circumference***, cm	34 (33-35)	34 (33.5-34.5)	34 (33.5-34.5)	1.00
**Balloon atrial septostomy**, N (%)	60 (58)	3 (9)	0 (0)	-
**Balloon atrial septostomy access route,** N (%)†<br>Femoral<br>Umbilical	35 (34)<br>23 (22)	3 (9)<br>0 (0)	-<br>-	-

**Preoperative ventilation**<br>N (%)<br>days	 67 (64)<br>2 (1-3)	 19 (54)<br>5 (4-8)	 12 (29)<br>6 (2-9)	 <br>0.101
**Preoperative resuscitation,** N (%)	1 (1)	2 (6)	0 (0)	-
**Preoperative inotropes**, N (%)	30 (29)	6 (17)	6 (15)	0.238
**Preoperative intensive care stay,** days	4 (2-6)	7 (5-9)	3 (2-7)	**0.001**

Continuous data are presented as median (25-75 centiles).

Continuous variables were compared using Kruskal-Wallis H test, categorical variables were compared with Chi-Squared or Fisher's Exact test.

p_{FDR} in bold are significant

Birth weight Z-scores were calculated with the UK-WHO reference data

*Missing ($\geq$5%): Apgar score 5-minutes N=11; arterial cord pH at birth N=67; head circumference N=80

†unknown Balloon Atrial Septostomy access route N=2.

**Table S2. Perioperative clinical characteristics of infants who underwent pre and postoperative MRI by CHD subgroup**				
	**Transposition of the great arteries N=88**	**Single Ventricle Physiology N=28**	**Left ventricular outflow tract and/or aortic arch obstruction N=30**	p_{FDR}
**Age at postoperative scan**, days	22 (16-30)	21 (15-29)	22 (18-36)	0.732
**Postmenstrual age at postoperative scan**, weeks	42.9 (41-6-44.1)	42.1 (40.9-43.4)	42.7 (41.0-43.6)	0.256
**Time preoperative MRI to surgery**, days	4 (2-7)	3 (1-4)	4 (2-6)	0.086
**Time surgery to postoperative MRI**, days	10 (7-14)	11 (8-15)	8 (6-12)	0.086
**Time preoperative to postoperative MRI**, days	14 (11-22)	17 (10-21)	13 (10-20)	0.764
**Male**, N (%)	57 (65)	20 (71)	22 (73)	0.727
**Gestational age at birth,** weeks	39.4 (38.6-40.4)	38.7 (38-39.3)	39.0 (38.2-40.1)	0.086
**Postnatal age at surgery**, days	10 (8-13)	7 (6-11)	11 (9-21)	**0.022**
**Postmenstrual age at surgery**, weeks	41.1 (40.1 -42.1)	39.9 (38.9 – 42.0)	41 (39.6 – 42.5)	0.047
**Type of Surgery,** N (%)				
Arterial switch operation	82	0	0	-
	3	0	0	-

Arterial switch operation with aortic arch repair	1	25	0	-
Norwood procedure	1	2	0	-
AP Shunt	1	0	0	-
BT Shunt	0	1	0	-
Aortic arch repair with pulmonary artery banding	0	0	24	-
Aortic arch repair	0	0	2	-
Aortic arch repair with aortic valve repair	0	0	1	-
Aortic arch repair with Ross-Konno procedure	0	0	1	-
Ross-Konno procedure	0	0	1	-
Hybrid procedure	0	0	1	-
Biventricular repair for hypoplastic left heart complex				
**Cardiopulmonary bypass†**				
N (%)	87 (99)	28 (100)	27 (90)	0.086
duration, minutes	172 (141-198)	172 (150 – 188)	133 (113 – 184)	0.064
**Aortic cross clamp time,** minutes	114 (94-130)	71 (57-100)	63 (37-94)	**<0.001**
**Selective cerebral perfusion**				
N (%)	6 (7)	25 (89)	19 (63)	**<0.001**
duration, minutes	27 (19-30)	34 (30-50)	40 (32-40)	0.064

**Lowest intraoperative temperature,** °C	28 (27-30)	20 (19-22)	20 (18.25-23.5)	**<0.001**
**ECMO or VAD,** N (%)	4 (5)	4 (14)	0 (0)	0.086
**Delayed sternal closure,** N (%)	37 (42)	19 (68)	11 (37)	0.064
**Renal replacement therapy,** N (%)	6 (7)	3 (11)	0 (0)	0.241
**Necrotizing enterocolitis,** N (%)	4 (5)	7 (25)	1 (3)	**0.013**
**Sepsis before postoperative MRI,** N (%)	6 (7)	6 (21)	5 (17)	0.086
**Seizures,** N (%)	3 (3)	3 (11)	2 (7)	0.241
**Postoperative resuscitation,** N (%)	1 (1)	0 (0)	0 (0)	-
**Postoperative mechanical ventilation duration**, days	3 (2-4)	9 (5-13)	3 (2-5)	**<0.001**
**Postoperative inotropes duration***, days	4 (3-5)	11 (7-15)	3 (1-5)	**<0.001**
**Non-cardiac interventions,** N (%)	7 (8)	2 (7)	2 (7)	1.00
**Postoperative intensive care stay**, days	6 (4-8)	15 (8-31)	6 (4-8)	**<0.001**
**Hospital stay**, days	23 (16-37)	62 (16-109)	25 (19-31)	**<0.001**
**30-day mortality after surgery**, N (%)	0 (0)	0 (0)	0 (0)	-
Continuous data are presented as median (25-75 centiles).				

Continuous variables were compared using Kruskal-Wallis H test, categorical variables were compared with Chi-Squared or Fisher's Exact test.<br>p_{FDR} in bold are significant<br>ECMO extracorporeal membrane oxygenation; VAD ventricular assist device<br>*Missing (≥5%): postoperative inotropes duration N=57.<br>†Surgical procedures without bypass: Arch repair for LVOTO N=2; Hybrid Procedure for LVOTO N=1; Missing data on bypass: A-P shunt for TGA N=1.

Table S3. Information on location of injuries

Injury type	Location information	Preoperative Injury N=180	New Postoperative Injury N=146
**White Matter Injury**	Side left/right/both, n (%)	14/10/20 (32/23/46)	6/12/15 (18/36/46)
	Lesion(s), No.	2 (1-5)	2 (1-5)
	Frontal, n (%)	24 (53)	32 (74)
	Parietal, n (%)	33 (73)	28 (65)
	Temporal, n (%)	7 (16)	4 (9)
	Occipital, n (%)	9 (20)	7 (16)
**Arterial Ischemic Stroke**	Side left/right/both	7/4/0 (64/36/0)	4/10/1 (27/67/7)
	Anterior cerebral artery, n (%)	1 (9)	0 (0)
	Middle cerebral artery, n (%)	6 (55)	4 (27)
	Perforator Branch, n (%)	4 (36)	11 (73)
	Posterior cerebral artery, n (%)	1 (9)	0 (0)
**Cerebral Sinus Venous Thrombosis**	Transverse, n (%)	-	11 (73)
	Straight, n (%)		2 (13)
	Transverse and jugular, n (%)		2 (13)

Table S4. Associations between clinical variables and new postoperative arterial ischemic stroke

	No new postoperative AIS (N=131)	New postoperative AIS (N=15)	p_{FDR}	No new postoperative AIS in infants who underwent CPB (N=127)	New postoperative AIS in infants who underwent CPB (N=15)	p_{FDR}
**CHD subgroup**, N (%)						
Transposition of the great arteries	84 (64)	4 (27)		83 (65)	4 (27)	
Single ventricle physiology	20 (15)	8 (53)		20 (16)	8 (53)	
Left ventricular outflow tract and/or aortic arch obstruction	27 (21)	3 (20)	**0.007**	24 (19)	3 (20)	**0.010**
**Gestational age at birth**, weeks	39.2 +/- 1.3	39.2 +/- 1.6	0.974	39.2 +/- 1.3	39.2 +/- 1.6	0.89
**Postnatal age at surgery**, days	11 (7-15)	7 (6-9)	**0.019**	11 (8-15)	7 (6-9)	**0.020**
**Cardiopulmonary bypass duration**, minutes	161 (130-194)	176 (143-188)	0.669	164 (136-195)	176 (143-188)	0.78
**Aortic cross clamp time,** minutes	102 (75-126)	88 (61-108)	0.269	103 (77-127)	88 (61-108)	0.21

**Selective cerebral perfusion**						
N (%)	38 (29)	12 (80)*	**0.003**	38 (30)	12 (80)*	**0.004**
minutes	35 (29-49)	37 (29-43)	0.941	35 (29-48)	37 (29-43)	0.90
**Lowest intraoperative temperature,** °C	27.4 (21.6-30.0)	19.7 (19.0-27.5)	**0.048**	27.0 (22.0-30.0)	19.7 (19.0-27.5)	0.056
**Delayed sternal closure,** N (%)	57 (44)	10 (67)	0.242	57 (45)	10 (67)	0.32
**Sepsis,** N (%)	14 (11)	3 (20)	0.561	14 (14)	3 (20)	0.54
**Postoperative mechanical ventilation duration**, days	3 (2-5)	6 (4-15)	**0.004**	3 (2-5)	6 (4-15)	**0.006**
**Postoperative intensive care stay**, days	6 (4-8)	11 (8-30)	**0.019**	6 (4-8)	11 (8-30)	**0.022**
**Time surgery to postoperative MRI**, days	9 (7-14)	10 (8-15)	0.669	9 (7-14)	10 (8-15)	0.68
**Age postoperative MRI**, days	22 (16-30)	20 (14-22)	0.298	22 (16-30)	20 (14-22)	0.32
**Postmenstrual age postoperative MRI,** weeks	42.7 (41.2-44.0)	42.2 (41.3-43.2)	0.581	42.8 (41.2-44.0)	42.2 (41.3-43.2)	0.54

**Postoperative cerebral sinus venous thrombosis,** N (%)	12 (9)	3 (20)	0.298	12 (9)	3 (20)	0.32

Continuous data are presented as mean +/- standard deviation when normally distributed, or as median (25-75 centiles) when not normally distributed.

***Hypoplastic left heart syndrome (n=8), hypoplastic aortic arch with aortic valve stenosis (n=1), hypoplastic aortic arch with aortic coarctation (n=1), hypoplastic left heart complex (n=1), transposition of the great arteries with hypoplastic aortic arch and ventricular septal defect (n=1).

p_{FDR} in bold are significant.

Table S5. Associations between clinical variables and preoperative arterial ischemic stroke

	No preoperative AIS (N=169)	Preoperative AIS (N=11)	p_{FDR}
**CHD subgroup**, N (%)			
Transposition of the great arteries	95 (56)	9 (82)	
Single ventricle physiology	33 (20)	2 (18)	
Left ventricular outflow tract and/or aortic arch obstruction	41 (24)	0 (0)	0.354
**Male**, N (%)	107 (63)	9 (82)	0.565
**Approach to labor**, N (%)			
Spontaneous vaginal	48 (29)	4 (36)	
Induced vaginal	49 (30)	3 (27)	
Elective cesarean	29 (18)	3 (27)	
Emergency cesarean	39 (24)	1 (9)	0.931
**Instrumental vaginal delivery, N (%)**	13 (15)	1 (14)	0.487
Ventouse	8 (8)	1 (14)	
Forceps	5 (6)	0 (0)	
**Birth at cardiac center**, N (%)	127 (75)	7 (64)	0.625
**Antenatal diagnosis**, N (%)	111 (66)	7 (64)	1.00
**Gestational age at birth**, weeks	39.1 +/- 1.3	38.5 +/- 1.4	0.402
**Apgar score 5-minutes***	9 (8-9)	8 (6-8)	**0.019**

**Balloon atrial septostomy**, N (%)	55 (32)	8 (73)	0.089
Femoral access[†]	34	4	
Umbilical access	19	4	0.354
Transposition of the great arteries	54/95 (57)	6/9 (67)	0.887
**Preoperative mechanical ventilation**			
N (%)	88 (52)	10 (91)	0.089
days	2 (1-5)	2.5 (1-6.75)	0.981
**Preoperative inotropes**, N (%)	36 (21)	6 (55)	0.089
**Preoperative intensive care stay,** days	4 (2-7)	6 (4-11)	0.199
**Age at preoperative MRI**, days	5 (3-8)	7 (3-8)	0.624
**Postmenstrual age at preoperative MRI**, weeks	39.7 (39.0-40.9)	39.7 (37.9-40.9)	0.624

Continuous data are presented as mean +/- standard deviation when normally distributed, or as median (25-75 centiles) when not normally distributed.

p_{FDR} in bold are significant

*Missing N=10/1

[†]Missing N=2/0

Table S6. Associations between clinical variables and preoperative white matter injury			
	**No preoperative WMI (N=135)**	**Preoperative WMI (N=45)**	**p_{FDR}**
**CHD subgroup**, N (%)			
Transposition of the great arteries	73 (54)	31 (69)	
Single ventricle physiology	30 (22)	5 (11)	
Left ventricular outflow tract and/or aortic arch obstruction	32 (24)	9 (20)	0.44
**Male**, N (%)	83 (62)	33 (73)	0.29
**Approach to labor**, N (%)			
Spontaneous vaginal	40 (30)	12 (27)	
Induced vaginal	34 (26)	18 (41)	
Elective cesarean	25 (19)	7 (16)	
Emergency cesarean	33 (25)	7 (16)	0.49
**Instrumental Vaginal Delivery**, N (%)	11 (8)	3 (7)	1.00
Ventouse	6 (4)	3 (7)	
Forceps	5 (4)	0 (0)	
**Birth at cardiac center**, N (%)	101 (75)	33 (73)	1.00
**Antenatal diagnosis**, N (%)	87 (64)	31 (69)	0.87
**Gestational age at birth**, weeks	39.1 +/- 1.32	38.7 +/- 1.3	0.42
**Apgar score 5-minutes***	9 (8-9)	9 (8-9)	1.00
**Balloon atrial septostomy**, N (%)	41 (30)	22 (49)	0.29
Femoral access[†]	26 (19)	12 (27)	0.44
Umbilical access	15 (11)	8 (18)	
Transposition of the great arteries	39/73 (52)	21/31 (68)	0.49

**Preoperative mechanical ventilation**			
N (%)	69 (52)	29 (64)	0.44
days	2 (1-5)	2 (1-4)	0.57
**Preoperative inotropes**, N (%)	33 (24)	9 (20)	0.87
**Preoperative intensive care stay,** days	4 (2-7)	4 (2-6)	0.57
**Age at preoperative MRI**, days	5 (3-7)	6 (4-9)	0.29
**Postmenstrual age at preoperative MRI**, weeks	39.9 (39.0-40.9)	39.6 (38.7-40.7)	0.57

Continuous data are presented as mean +/- standard deviation when normally distributed, or as median (25-75 centiles) when not normally distributed.

*Missing N=8/3

†Missing N=0/2

p_{FDR} in bold are significant

Table S7. Associations between clinical variables and new postoperative white matter injury						
	**No new postoperative WMI (N=103)**	**New postoperative WMI (N=43)**	**p_{FDR}**	**No new postoperative WMI in infants who underwent CPB (N=101)**	**New postoperative WMI a in infants who underwent CPB (N=41)**	**p_{FDR}**
**CHD subgroup**, N (%)						
Transposition of the great arteries	67 (65)	21 (49)		66 (65)	21 (51)	
Single ventricle physiology	14 (14)	14 (33)		14 (14)	14 (34)	
Left ventricular outflow tract and/or aortic arch obstruction	22 (21)	8 (18)	0.14	21 (21)	6 (15)	0.12
**Gestational age at birth**, weeks	39.3 +/- 1.3	38.8 +/- 1.4	0.14	39.3 +/- 1.3	38.9 +/- 1.4	0.18
**Postnatal age at surgery**, days	10 (7-14)	9 (7-13)	0.60	10 (8-14)	9 (7-13)	0.53
**Cardiopulmonary bypass duration**, minutes	165 (124-195)	159 (146-193)	0.92	166 (128-194)	164 (150-193)	0.63
**Aortic cross clamp time,** minutes	103 (75-129)	92 (70-114)	0.26	103 (76-129)	93 (74-114)	0.39
**Selective cerebral perfusion** N (%)	32 (31)	18 (42)	0.43	32 (32)	18 (44)	0.36

minutes	40 (30-51)	32 (29-44)	0.48	40 (30-50)	32 (29-44)	0.48
**Lowest intraoperative temperature,** °C	27.4 (21.5-30.0)	24.4 (19.6-28.0)	0.18	27 (21.8-30.0)	23.5 (20.0-28.0)	0.18
**Delayed sternal closure,** N (%)	47 (46)	20 (47)	1.00	47 (47)	20 (49)	0.85
**Sepsis,** N (%)	10 (10)	7 (16)	0.43	10 (10)	7 (17)	0.42
**Postoperative mechanical ventilation duration**, days	3 (2-6)	4 (3-7)	0.14	3 (2-6)	4 (3-7)	0.12
**Postoperative intensive care stay**, days	6 (4-8)	6 (4-13)	0.85	6 (4-8)	6 (4-13)	0.54
**Time surgery to postoperative MRI**, days	10 (8-17)	8 (7-13)	0.14	10 (8-16)	9 (7-13)	0.24
**Age postoperative MRI**, days	22 (17-34)	20 (14-28)	0.14	22 (17-33)	20 (15-28)	0.24
**Postmenstrual age postoperative MRI,** weeks	43.1 (41.3-44.2)	42.0 (40.8-43.3)	0.065	43.1 (41.3-44.1)	42.0 (41.1-43.3)	0.12
**Preoperative MRI white matter injury**, N (%)	24 (23)	14 (33)	0.48	23 (23)	13 (32)	0.42

Continuous data are presented as mean +/- standard deviation when normally distributed, or as median (25-75 centiles) when not normally distributed.

p_{FDR} in bold are significant.

Table S8. Associations between clinical variables and new postoperative cerebral sinus venous thrombosis						
	**No new postoperative CSVT (N=131)**	**New postoperative CSVT (N=15)****	**p_{FDR}**	**No new postoperative CSVT in infants who underwent CPB (N=127)**	**New postoperative CSVT in infants who underwent CPB (N=15)****	**p_{FDR}**
**CHD subgroup**, N (%)						
Transposition of the great arteries	77 (59)	11 (74)		76 (60)	11 (74)	
Single ventricle physiology	26 (20)	2 (13)		26 (20)	2 (13)	
Left ventricular outflow tract and/or aortic arch obstruction	28 (21)	2 (13)	0.89	25 (20)	2 (13)	0.97
**Gestational age at birth**, weeks	39.2 +/- 1.3	38.7 +/- 1.5	0.62	39.3 +/- 1.3	38.7 +/- 1.5	0.64
**Postnatal age at surgery**, days	10 (7-14)	10 (7-12)	0.89	10 (7-14)	10 (7-12)	0.97
**Cardiopulmonary bypass duration**, minutes	163 (137-192)	175 (122-209)	0.98	164 (138-192)	175 (122-209)	0.97
**Aortic cross clamp time,** minutes	100 (69-119)	107 (82-148)	0.62	100 (74-121)	107 (82-148)	0.64
**Selective cerebral perfusion**						

N (%)	44 (34)	6 (40)	0.97	44 (35)	6 (40)	0.97
minutes	35 (29-50)	39 (28-45)	0.98	35 (30-49)	39 (28-45)	1.00
**Lowest intraoperative temperature,** °C	27 (20-30)	24 (22-28)	0.79	27 (20-30)	24 (22-28)	0.70
**Delayed sternal closure,** N (%)	57 (44)	10 (67)	0.62	57 (45)	10 (67)	0.64
**Sepsis,** N (%)	16 (12)	1 (20)	1.00	16 (16)	1 (7)	1.00
**Postoperative mechanical ventilation duration**, days	3 (2-6)	4 (3-6)	0.79	4 (3-6)	4 (3-6)	0.70
**Postoperative intensive care stay**, days	6 (4-8)	8 (4-10)	0.89	6 (4-9)	8 (4-10)	0.97
**Time surgery to postoperative MRI**, days	10 (7-15)	9 (7-13)	0.89	10 (7-15)	9 (7-13)	1.00
**Age postoperative MRI**, days	22 (16-30)	20 (14-28)	0.79	22 (16-30)	20 (14-28)	0.77
**Postmenstrual age postoperative MRI,** weeks	42.7 (41.3-44.0)	42.3 (40.5-43.5)	0.62	42.9 (41.3-44.0)	42.3 (40.5-43.5)	0.64

Continuous data are presented as mean +/- standard deviation when normally distributed, or as median (25-75 centiles) when not normally distributed.

**Transposition of the great arteries with intact ventricular septum (n=8), transposition of the great arteries with hypoplastic aortic arch and ventricular septal defect (n=1), transposition of the great arteries with ventricular septal defect (n=1), double outlet right ventricle with transposition of the great arteries, aortic coarctation and ventricular septal defect (n=1), unbalanced atrioventricular septal defect (n=1), hypoplastic left heart syndrome (n=1), hypoplastic aortic arch with aortic valve stenosis (n=1), aortic coarctation (n=1)

p_{FDR} in bold are significant.

Table S9. Clinical characteristics of infants by approach to labor					
	**Spontaneous Vaginal Delivery (N=52)**	**Induced Vaginal Delivery (N=52)**	**Elective Cesarean Section (N=32)**	**Emergency Cesarean Section (N=40)**	**p-value**
**Gestational age at birth**, weeks	39.7 (39.1-40.4)	38.7 (38.4-39.4)	38.4 (38.0-39.0)	38.9 (37.6-40.2)	**0.001***
**Birth weight**					
Grams	3460 (3000-3911)	3140 (2888-3409)	3215 (2893-3432)	3035 (2871-3665)	
Z-score	0.1 (-0.7-0.9)	-0.9 (-1.7-0)	-0.4 (-1.0-0.3)	-0.3 (-0.8-0.2)	0.087
**Head circumference**, cm	35 (34-36)	34 (33-35)	34 (33-35)	34 (33.5-34.5)	0.272
**Male**, N (%)	36 (69)	30 (58)	19 (59)	28 (70)	0.486
**Antenatal diagnosis**, N (%)	25 (48)	45 (87)	20 (63)	26 (65)	**<0.001****
**Birth at cardiac center**, N (%)	30 (58)	45 (87)	27 (84)	30 (75)	**0.004**
**CHD subgroup**, N (%)					
TGA	35 (67)	28 (54)	15 (47)	25 (63)	

SVP<br>LVOTO	6 (12)<br>11 (21)	14 (27)<br>10 (19)	8 (25)<br>9 (28)	6 (15)<br>9 (22)	 0.362
**Twin**, N (%)	0 (0)	1 (2)	4 (13)	1 (3)	-
**Apgar score 5-minutes**	9 (8-9)	9 (9-9)	9 (8-9)	9 (8-9)	0.584
**Arterial cord pH at birth**	7.26 (7.18-7.33)	7.30 (7.22-7.38)	7.29 (7.27-7.32)	7.29 (7.25-7.34)	0.770
**Induced for additional non-CHD reasons *****		13			
Gestational diabetes		4			
Cholestasis of pregnancy		2			
Pregnancy induced hypertension		2			
Fetal age		2			
Potential chorioamnionitis		1			
Pathological CTG		1			
Fetal macrosomia		1			

Continuous data are presented as median (25-75 centiles).

Birth weight Z-scores were calculated with the UK-WHO reference data

*Gestational age at birth differences by approach to labor: spontaneous vaginal vs induced vaginal ($p<0.001$), spontaneous vaginal vs elective cesarean ($p<0.001$), spontaneous vaginal vs emergency cesarean ($p=0.025$), induced vaginal vs elective cesarean ($p=0.054$), induced vaginal vs emergency cesarean (0.994), elective cesarean vs emergency cesarean ($p=0.208$).

**Rate of antenatal diagnosis differences by approach to labor: spontaneous vaginal vs induced vaginal ($p<0.001$), spontaneous vaginal vs elective cesarean ($p=0.288$), spontaneous vaginal vs emergency cesarean ($p=0.159$), induced vaginal vs elective cesarean ($p=0.022$), induced vaginal vs emergency cesarean ($p=0.029$), elective cesarean vs emergency cesarean ($p=1.00$).

*** Missing data N=2

p_{FDR} in bold are significant

Table S10. The relationship between new postoperative AIS, selective cerebral perfusion and lowest intraoperative temperature

	**No SCP (N=95)**		**SCP with mild hypothermia (N=2)**		**SCP with moderate hypothermia (N=14)**		**SCP with deep hypothermia (N=34)**	
	No AIS	AIS (right)	No AIS	AIS (right)	No AIS	AIS (right)	No AIS	AIS (right)
**TGA**	78	3 (2)	0	0	4	0	1	1 (1)
**SVP**	3	0	0	1 (0)	7	0	10	7 (5)
**LVOTO**	11	0	0	1 (1)*	3	0	13	2 (2)

*New postoperative AIS in both hemispheres.

Table S11. Brain Imaging findings in infants with new postoperative cerebral sinus venous thrombosis

	**Preoperative Brain Imaging Findings**	**New Postoperative Brain Imaging Findings**
**1**	WMI, subdural hemorrhage, cerebellar hemorrhage	CSVT, AIS, WMI
**2**	WMI, cerebellar hemorrhage	CSVT, subdural hemorrhage
**3**	-	CSVT, intraventricular hemorrhage grade 2, subdural hemorrhage
**4**	Hypoxic-ischemic watershed injury	CSVT, AIS, WMI
**5**	WMI, intraventricular hemorrhage grade 2	CSVT, AIS, hypoxic-ischemic watershed injury
**6**	AIS, intraventricular hemorrhage grade 1	CSVT

**7**	-	CSVT, intraparenchymal hemorrhage
**8**	subdural hemorrhage	CSVT, WMI
**9**	WMI	CSVT, WMI, intraventricular hemorrhage Grade 1
**10**	WMI, subdural hemorrhage	CSVT
**11**	-	CSVT, subdural hemorrhage
**12**	-	CSVT
**13**	-	CSVT, subdural hemorrhage
**14**	AIS, WMI, subdural hemorrhage	CSVT, WMI
**15**	-	CSVT, WMI
For details of image review please see Stegeman et al 2021, A Uniform Description of Perioperative Brain MRI Findings in Infants with Severe Congenital Heart Disease: Results of a European Collaboration		

Table S12. Significant predictors of new postoperative injuries according to logistic regression analyses in infants who underwent cardiopulmonary bypass

Model	Odds ratio (95% CI)	P-value
**New white matter injury**		
Single ventricle physiology	2.97 (1.21-7.32)	0.017
Younger postmenstrual age at preoperative MRI	1.01 (0.94-1.39)	0.009
**New arterial ischemic stroke**		
Selective Cerebral Perfusion*ǂ	9.54 (2.73-44.98)	0.001
Younger postnatal age at surgery*	1.18 (1.05-1.33)	0.022
**New cerebral sinus venous thrombosis**		
Transposition of the great arteries	13.07 (2.01-97.71)	0.009
Lower minimum intraoperative temperature*	1.20 (1.03-1.35)	0.026
Delayed sternal closure*	3.69 (1.14-13.79)	0.037
Younger postmenstrual age at postoperative MRI	1.26 (0.99-1.48)	0.075

*Associated with CHD subgroup.

ǂ When including SVP in the final model, SVP was not a significant risk factor (OR=1.32, 95% CI 0.32-5.43, p=0.699) and younger postnatal age at surgery (p=0.033) and selective cerebral perfusion (p=0.008) remained risk factors for new postoperative AIS.
